# Genetic Control of Grain Protein and Gluten Content: Winter vs. Spring Wheat

**DOI:** 10.3390/ijms262211159

**Published:** 2025-11-18

**Authors:** Antonina A. Kiseleva, Anna V. Fedyaeva, Irina N. Leonova, Elena A. Salina

**Affiliations:** 1 The Federal Research Center, Institute of Cytology and Genetics SB RAS, Novosibirsk 630090, Russia; fedyaevaav@bionet.nsc.ru (A.V.F.); leonova@bionet.nsc.ru (I.N.L.); salina@bionet.nsc.ru (E.A.S.); 2 Kurchatov Genomics Center, Institute of Cytology and Genetics SB RAS, Novosibirsk 630090, Russia

**Keywords:** *Triticum aestivum* L., grain protein content, association mapping, GWAS, spring and winter wheat

## Abstract

Common wheat breeding programs prioritize the development of high grain protein content (GPC) varieties, as GPC influences milling efficiency and end-use quality. However, the molecular basis of protein and gluten accumulation in wheat grains remains insufficiently understood, particularly regarding genetic differences between spring and winter types. We analyzed 170 winter wheat varieties from diverse domestic and international breeding programs cultivated in the European part of Russia over two growing seasons. Genome-wide association study identified 26 markers linked to GPC and 23 to gluten content (*p* < 0.001), with the strongest associations on chromosomes 4A and 2D. Variation in *NAM-A1* also significantly affected GPC: varieties with the *NAM-A1d* allele showed lower protein content than *NAM-A1a/c* carriers (*p* < 0.01). We combined associations identified here with our previous GWAS results for GPC in spring wheat and further compared them with 17 additional studies including both spring and winter varieties. This analysis highlighted loci on chromosomes 3DL, 5AL, and 6AS (confirmed in at least two previous studies) for marker-assisted selection to improve grain quality. The distribution of loci showed no clear distinction between spring and winter wheat, suggesting that, despite environmental and developmental differences, the genetic basis of protein accumulation is largely shared.

## 1. Introduction

Grain protein content (GPC) and gluten concentration in wheat are crucial traits influencing nutritional value and baking properties. Wheat protein is one of the main sources of energy and nutrients for the human diet. These traits also play a key role in processing quality, providing texture, elasticity and extensibility of dough (dependent on gluten quantity and quality) [1].

GPC refers to the accumulation of nitrogenous compounds, primarily storage proteins including albumins, globulins, gliadins, and glutenins within the wheat grain during development [2]. This quantitative trait is controlled by multiple genes and significantly influenced by environmental factors such as nitrogen availability in the soil, temperature, moisture, and sunlight during critical growth stages, especially grain filling [3,4]. Therefore, improving GPC through breeding and management is challenging due to its complex genetic control, significant environmental influence, and often inverse relationship with grain yield [5].

Both genetic mapping studies using biparental populations and genome-wide association studies (GWAS) have identified numerous Quantitative Trait Loci (QTLs) associated with GPC across all wheat chromosomes [5,6,7]. GPC is governed by genes involved in various metabolic pathways, including storage protein synthesis, nitrogen uptake, assimilation, transport, and grain development. The major components of grain protein are gluten storage proteins (gliadins and glutenins), which constitute 70–80% of the total grain protein [8]. Genes encoding these proteins are primarily located on the short arms of homoeologous group 1 chromosomes (1A, 1B, 1D) for high-molecular-weight (HMW) and low-molecular-weight (LMW) glutenin subunits, and on the short arms of group 6 chromosomes (6A, 6B, 6D) for gliadins [9,10]. Genes encoding transporters (e.g., nitrate transporters, ammonium transporters), enzymes involved in nitrogen assimilation (e.g., nitrate reductase, nitrite reductase, glutamine synthetase), and genes controlling nitrogen remobilization from vegetative tissues to the developing grain play a crucial role [11,12].

It is well established that grain protein content (GPC) in wheat is negatively correlated with yield, a relationship influenced by both genetic and environmental factors. Among the key environmental factors are nitrogen deficiency in the soil, insufficient nitrogen uptake during plant development, and low remobilization of nitrogen from vegetative tissues to the grain [13,14]. A particularly well-studied and important locus for increasing GPC in wheat is *Gpc-B1* (*NAM-B1*), located on chromosome 6B [15]. This gene, originating from *Triticum dicoccoides*, encodes a NAC transcription factor (*NAM-B1*) that accelerates senescence and nutrient remobilization from leaves to developing grains, thereby enhancing GPC [16]. Introgression of functional *NAM-B1* into modern wheat varieties has been associated with increased protein and micronutrient content, but it is rarely used due to its substantial negative impact on yield [16,17]. However, the reduction in thousand-grain weight is compensated by increases in other yield components, such as the number of grains per spike and productive tillering, resulting in no overall yield penalty [18]. Two other genes, *NAM-A1* and *NAM-D1*, have been mapped to chromosomes 6A and 6D, respectively, with *NAM-A1* presumed to perform a similar function to *NAM-B1* [19,20]. The effect of *NAM-A1* on yield and its components has not been thoroughly studied; however, results obtained from introgression lines of bread wheat and Russian cultivars carrying different *NAM-A1* alleles indicate no significant differences in grain weight per spike or thousand-grain weight [21,22].

On average, winter wheat tends to have lower GPC compared to spring wheat [23,24]. This difference is not solely genetic but is a complex interplay of genetic potential, growth cycle, environmental conditions, and historical breeding priorities. Winter wheat is sown in autumn, overwinters, and is harvested in summer. This longer growth cycle, particularly the extended vegetative phase, allows for greater biomass accumulation and often higher yield potential compared to spring wheat, which is sown and harvested within a single spring-to-summer season [25,26]. While winter wheat can take up nitrogen both in the autumn and spring, the remobilization of this nitrogen to the grain can be less concentrated or efficient relative to the larger biomass and starch accumulation, leading to a dilution effect [12,26,27]. Spring wheat, with its shorter, more intense growth period, often has a greater proportion of nitrogen uptake occurring closer to the anthesis stage and during grain-filling period, potentially leading to higher GPC [28,29].

The aims of this study were (1) to perform a genome-wide association study (GWAS) to map MTAs and identify genes involved in the accumulation of protein and gluten in winter wheat grain; and (2) to conduct a comparative analysis of the genetic control of protein and gluten content in common wheat with different growth habits (spring/winter).

## 2. Results

### 2.1. Evaluation of Grain Protein and Gluten Content

**Figure 1:**
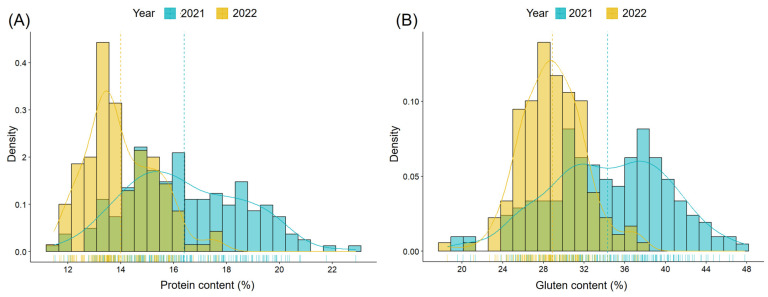
Distribution of winter wheat varieties grown in 2021 and 2022 by grain protein content ((**A**), blue for 2021; yellow for 2022) and gluten content ((**B**), blue for 2021; yellow for 2022). The green color represents the overlap of the two distributions. The vertical dashed lines indicate the mean values for each year, and the solid colored lines represent the corresponding density plots.

Grain protein content in 2021 ranged from 11.52% to 22.89%, and in 2022 from 11.47% to 17.68% (Figure 1, Table S1). Gluten content (GC) in 2021 ranged from 19.55% to 47.82%, and in 2022 from 18.54% to 37.33% (Figure 1, Table S1). On average, both protein and gluten content were lower in 2022 compared to 2021. Additionally, the variability of both traits was lower in 2022 than in 2021. In all cases, trait distributions were approximately normal, though in 2021 they were more flattened for both traits.

ANOVA revealed that both GPC and GC differed significantly (*p*-value < 0.0001) among genotypes, environmental conditions, and genotype × environment interactions (Table S2). GPC and GC were significantly correlated (r^2^ = 0.92, *p*-value < 0.0001) (Figure S1), both on average and in each year individually. The correlation coefficient was slightly lower in 2022 (r^2^ = 0.76, *p*-value < 0.0001) than in 2021 (r^2^ = 0.92, *p*-value < 0.0001). The heritability of the studied traits was high both for protein (0.80) and for gluten (0.69) content, which suggests a substantial genetic contribution to the variation in these traits.

Grain protein content above 15% in common wheat is indicative of high quality according to interstate standard GOST 9353-2016 [30]. In 2021, the highest protein content was observed in the varieties Mikhaylovka (20%) and Eritrospermum 59 (21%). The lowest value was recorded for the variety Tau (10%). A high gluten content (>28%) was observed in 129 varieties, with the highest value found in Eritrospermum 59 (44%) and the lowest (18%) in the varieties Tau and Rifey.

In 2022, 35 of the studied varieties exceeded the 15% threshold for protein content. The highest protein content was recorded in the variety Donka (17.7%), and the lowest in Soraja (11.5%). Analysis of gluten content in 2022 showed that 41 varieties had a high level (>35%). The highest value was observed in the variety Banatka (39.0%), while the lowest was found in Shatilovskaya (18.5%).

### 2.2. Allelic Diversity of the NAM-A1 Gene and Its Effect on Grain Protein and Gluten Content

In a collection of winter bread wheat varieties, the allelic diversity of *NAM-B1* and *NAM-A1* genes—known to influence grain protein content—was investigated.

Currently, three variants of the *NAM-B1* gene are known: a functional wild-type allele (rarely found in modern varieties); a non-functional allele caused by a single-nucleotide insertion in exon 1; a complete gene deletion (~200 kb in length). Analysis showed that all varieties in the studied panel carried the same non-functional *NAM-B1* allele.

The distribution of *NAM-A1* alleles among the winter wheat varieties is presented in Figure S2. The most common allele was *NAM-A1c* (71% of varieties), followed by *NAM-A1d* (16%), while the rarest was *NAM-A1a* (7%). The *NAM-A1b* allele (corresponding to haplotype C/DEL) was not detected in this panel.

**Figure 2:**
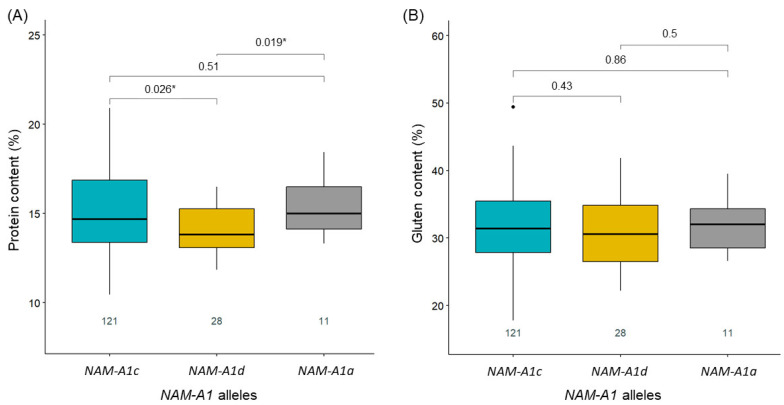
Boxplots showing grain protein content (**A**) and gluten content (**B**) in winter common wheat varieties depending on the *NAM-A1* allele. The significance level indicated by * corresponds to *p*-values < 0.05. The horizontal line inside each box represents the median value. Numbers at the bottom indicate the number of genotypes in each group. Blue color corresponds to *NAM-A1c* allele, yellow to *NAM-A1d* allele and grey to *NAM-A1a* allele. The black dots represent outliers in the box plot.

Grain protein content was significantly higher (*p* < 0.05) in varieties carrying the *NAM-A1a* and *NAM-A1c* alleles compared to those with *NAM-A1d* (Figure 2, Table S3). However, no significant association was observed between *NAM-A1* alleles and gluten content (Figure 2, Table S3).

### 2.3. Genome-Wide Association Study for Grain Protein and Gluten Content in Winter Wheat

**Figure 3:**
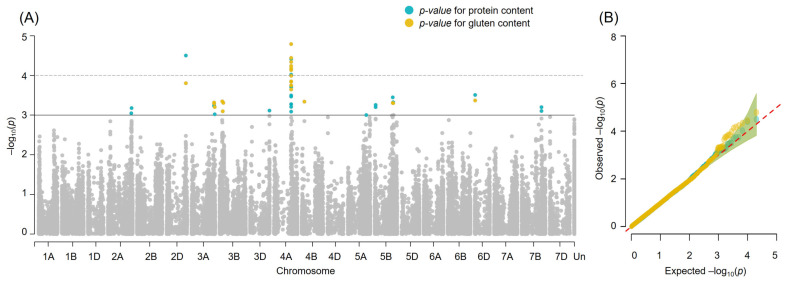
Summary of Genome-Wide Association Study (GWAS) Results for Grain Protein and Gluten Content in Winter Bread Wheat. Blue points indicate SNPs associated with grain protein and yellow those associated with gluten contents. (**A**) Manhattan plot based on the MLM model. The horizontal solid line indicates the threshold of significance *p* < 0.001. The horizontal dashed line indicates the threshold of significance *p* < 0.0001. The grey dots represent markers below the significance threshold; (**B**) Quantile–quantile (QQ) plots showing observed versus expected −log_10_ (*p*) values. X-axis: expected −log10 (*p*) values. Y-axis: observed −log10 (*p*) values. The red dotted line represents the standard expected relationship among markers, and the different colored shaded areas indicate the 95% confidence interval for the QQ plot under the null hypothesis.

A genome-wide association study (GWAS) using a mixed linear model (MLM) identified 26 SNP markers significantly associated with grain protein content (*p* < 0.001) and 23 markers significantly associated with gluten content (*p* < 0.001). These markers were distributed across chromosomes 2A, 2D, 3A, 3B, 4A, 4B, 5A, 5B, 6D, and 7B (Figure 3, Table 1). Most of the identified markers were significantly associated with both traits—protein and gluten content. The estimated effects of individual markers ranged from −0.96% to +1.21% for protein content and from −2.54% to +3.29% for gluten content.

The most significant locus associated with both traits is located on chromosome 4A, including 11 significant markers for grain protein content and 12 for gluten content (Table 1), spanning a 1.440.917 bp interval. Within this region, 13 genes are located. Expression pattern analysis revealed that the gene *TraesCS4A02G235900* is specifically expressed in the grain from the hard dough stage through senescence. This gene encodes serpin domain-containing protein.

The second most significant GPC-associated locus is located on chromosome 2D and is marked by Excalibur_c44325_339, which falls within the coding region of the gene *TraesCS2D02G517200*. This gene encodes a glycerol kinase and is primarily expressed after flowering in the flag leaf, the fifth leaf, and the flag leaf sheath.

### 2.4. Candidate Genes for Loci Supported by Three or More Studies

Three high-confidence loci associated with grain protein content were identified. In addition to being detected in the present study, these loci were consistently reported in at least two independent investigations, confirming their reliability. The loci were mapped to the long arms of chromosomes 3D and 5A, and to the short arm of chromosome 6A, highlighting their potential relevance in the genetic control of protein accumulation in wheat grain. Five genes were identified on chromosome 3D, one on 5A, and nine on 6A. Their list, along with functional descriptions, is presented in Table 2.

## 3. Discussion

This study evaluated a diverse collection of common wheat (*Triticum aestivum* L.) varieties, consisting of 170 winter wheat varieties from various regions of the world, the majority of which were developed via Russian breeding programs. According to the interstate standard GOST 9353-2016, which defines wheat quality classes in the Russian Federation, grain protein contents (GPCs) of 15% or higher are classified as high-class wheat. Therefore, this threshold was used in our study to identify high-protein genotypes. This threshold was met by 69 varieties included in the present study, based on the average values of this trait over two years of field trials. Among the studied genotypes, several cultivars demonstrated consistently high grain protein content (GPC) across both years of field testing. These include Donka (originating from the Rostov region), Staritsinskaya (Tomsk region), and Banatka (Stavropol region). These cultivars can be recommended as promising donors of favorable alleles for improving grain protein content in future wheat breeding programs.

Previous studies have reported a broad range of heritability estimates for grain protein content, reflecting the strong dependence of this trait on both population structure and environmental conditions. While most reports highlight a pronounced and significant influence of the environment, GPC heritability is generally moderate to high, thereby supporting the applicability of genomic prediction and selection [5,7]. In our study, the broad-sense heritability of GPC was estimated at 0.80, which aligns with previous reports for winter wheat. For instance, heritability estimates in European winter wheat collections [31] and Chinese winter wheat collections [32] were similarly high, ranging from 0.72 to 0.93.

The *NAM-A1* gene has been implicated in the regulation of grain protein content, leaf senescence, and the duration of grain maturation [19,33,34,35]. Varieties carrying *NAM-A1a* generally exhibit the highest GPC, those with *NAM-A1c* display intermediate levels, and those with *NAM-A1d* the lowest [35]. Our results are consistent with these findings: the highest protein content was observed in varieties carrying the *NAM-A1a* allele, whereas the lowest values were associated with *NAM-A1d*. Notably, the *NAM-A1a* allele, despite its association with increased grain protein content, was relatively uncommon in our panel, being present in only 6% of the varieties.

### 3.1. Comparative Analysis of Loci Associated with Grain Protein Content in Winter and Spring Wheat

In this study, using GWAS, we identified 26 markers significantly associated (*p*-value < 0.001) with grain protein content and 23 markers significantly associated (*p*-value < 0.001) with gluten content. These markers were located on chromosomes 2A, 2D, 3A, 3B, 4A, 4B, 5A, 5B, 6D, and 7B.

To compare the obtained data with previously reported markers and loci primarily associated with grain protein content, we summarized information from 15 publications describing GWAS or QTL analyses conducted in spring wheat [36,37,38,39,40] and winter wheat [31,32,41,42,43,44,45,46,47,48], two studies on grain protein content in *T. durum* [49,50], and our two previous studies on spring wheat [21,51] (Table S4). Additionally, well-known genes such as *NAM-1*, *NAM-2*, gliadins and glutenins were included.

**Figure 4:**
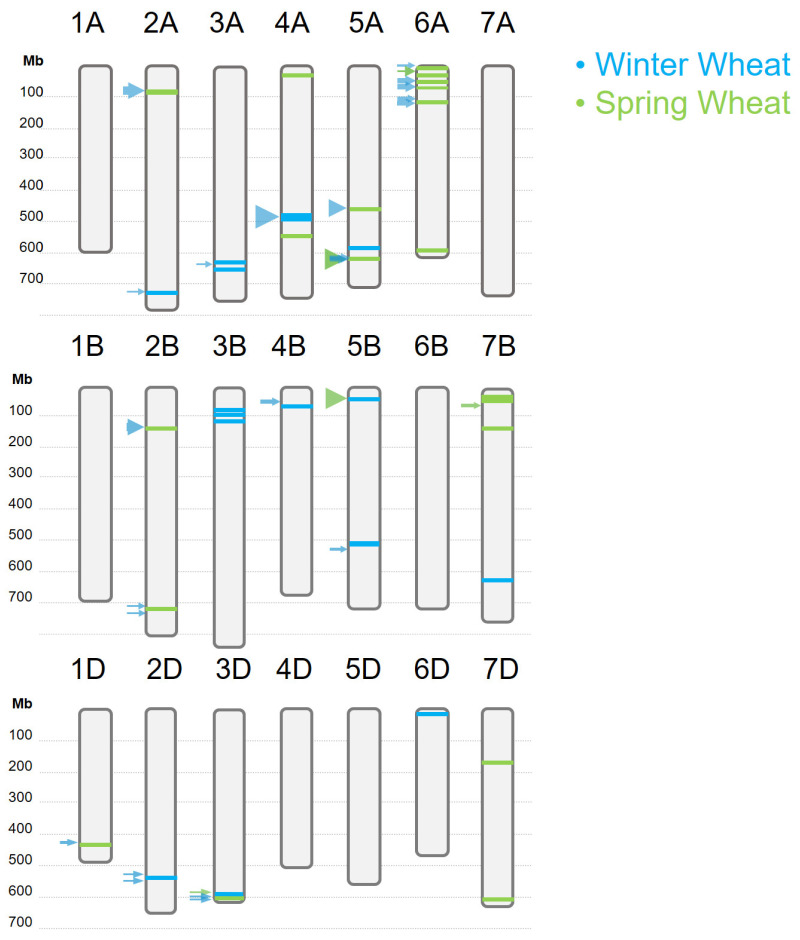
MTAs and QTLs identified in the present study in winter wheat, as well as in our previous studies on spring wheat [21,51], are shown as bars along the chromosomes. Arrows beside the chromosomes indicate the nearest or overlapping MTAs and QTLs reported by other authors. Arrow size corresponds to the number of underlying markers (the more markers, the larger the arrow). The green lines and arrows correspond to spring wheat loci, and the blue lines and arrows corre-spond to winter wheat.

In total, 639 marker–trait associations (MTAs) were incorporated into the analysis, including those identified in the present study (Table S5). Table S6 and Figure 4 present the markers from our studies (the current work on winter wheat and two earlier studies on spring wheat) that overlap with, or are located in close proximity to, previously reported MTAs for grain protein content.

On the long arm of chromosome 2A, three loci previously described in winter wheat were localized between two MTAs identified in our study. Two of these loci were reported by Voss-Fels et al. [45] and one by Geyer and Mohler [46]. On the long arm of chromosome 2D, two SNPs detected by Yang et al. [32], also in winter wheat, flanked the marker Excalibur_c44325_339 identified in the present study. It should be noted that the loci on the long arms of chromosomes 2A and 2D were syntenic (Figure S3).

On chromosome 3A, an SNP previously reported by Kartseva et al. [47] as associated with grain protein content in winter wheat was located between two closely positioned MTAs identified in this study. On the short arm of chromosome 3B, the locus identified here, consisting of three SNPs, was positioned between two loci previously reported in winter wheat [44,45].

On the long arm of chromosome 3D, the marker Excalibur_c17654_1090 identified in the present study in winter wheat was located near the TA015516-0532, which we previously reported as associated with gluten content in a spring wheat panel [51]. Between these two markers, two additional loci described in winter wheat were found [41,47]. Furthermore, immediately downstream of Excalibur_c17654_1090, another locus was reported by [45], also in winter wheat.

The locus on chromosome 4A (RefSeq v1.1: 544 177 849–545 618 766), consisting of 12 SNPs, overlapped with a locus previously identified in a population of RILs derived from a cross between two *T. durum* varieties, Duilio and Avonlea [49]. In that study, Marcotuli et al. did not specify whether these varieties were winter or spring types. However, Basso et al. reported autumn sowing of Duilio in September, suggesting that the studied population likely represented a winter growth habit [52]. The locus on chromosome 5BS also overlapped with a locus previously identified in a durum population of doubled haploid (DH) lines derived from the cross between Pelissier and Strongfield [50]. The growth habit of the parental varieties was not specified in that publication either; however, according to the Canadian variety registration database [53], Strongfield is a spring type, and therefore we infer that this population most likely represented a spring growth habit. Among genes within this locus on 4A we identified *TraesCS4A02G235900* (encodes serpin domain-containing protein), which is specifically expressed in the grain from the hard dough stage through senescence. The serpin gene family plays an essential role in cereal grain development and quality. Early studies identified over 20 serpin genes in barley, wheat, rye, and oats, following the discovery of a barley grain serpin functioning both as a storage protein during grain filling and as a major source of lysine, an essential amino acid [54,55]. In wheat, at least six serpins exhibiting inhibitory activity toward chymotrypsin- and cathepsin-like proteases have been characterized [56]. These serpins are thought to protect grain storage proteins from degradation by endogenous or exogenous proteases, particularly those targeting proline- and glutamine-rich substrates such as prolamins and glutenin subunits, which are crucial for grain nitrogen content and bread-making quality [56,57,58].

On the short arm of chromosome 4B, the SNP Kukri_c48199_102 identified in this study was located adjacent to a locus reported by Voss-Fels et al. [45] in winter wheat. On chromosome 5B, two loci were identified in our study, one on the short arm and one on the long arm. The markers AX-110033497 and wsnp_Ex_c8962_14947544 on the short arm overlapped with a locus previously reported in *T. durum* by Ruan et al. [50]. On the long arm, the SNPs RAC875_rep_c102342_470 and TA001138-0446 were located between two loci previously identified in winter wheat [45,46]. It is also worth noting the SNP AX-94647124 (RefSeq v1.1: 9 358 293) on chromosome 6D, which was located in proximity to *NAM-D1* (*TRAESCS6D02G096300*, RefSeq v1.1: 60 486 167).

Thus, the majority of the MTAs identified in this study overlapped with or were located in close proximity to previously reported MTAs or QTLs. In particular, the MTAs on chromosomes 2A, 2D, 3A, 3B, 4B, and 5BL corresponded to loci that had also been reported in winter wheat. The only locus previously identified in spring wheat that was located near one of the loci detected in the present study on winter wheat was on the long arm of chromosome 3D. This locus, also reported in our earlier work, was associated with grain gluten content [51].

When comparing the MTAs previously identified in our studies on a collection of spring wheat varieties [21,51] with loci reported in the literature, no similar pattern was observed; in contrast to winter wheat, the majority of loci identified in spring wheat did not correspond to loci reported in wheat of the same growth habit.

Most of the MTAs were located on the short arm of chromosome 6A. Four of these MTAs either mapped close to previously reported loci—for example, *QGpc.icg-6A.1* (Tdurum_contig63703_1143 and wsnp_Ra_c3996_7334169) was located immediately downstream of SNP AX-95186193 identified by Krishnappa et al. [43]—or overlapped with known loci, such as BS00065076_51, which fell within the locus described by Mahjourimajd et al. [38] and delimited by markers Kukri_c42078_708 and Kukri_c11106_292. Similarly, *QGpc.icg-6A.2* (Kukri_rep_c68344_627 and wsnp_CAP7_c1839_908011) overlapped with a locus defined by JD_c8888_741 and Kukri_c22149_276 and reported by Voss-Fels et al., while *QGpc.icg-6A.3* (tplb0032i10_420 and BobWhite_c20782_697) intersected with two loci also described by Voss-Fels et al. [45]. Except for the locus described by Mahjourimajd et al. [38] between Kukri_c42078_708 and Kukri_c11106_292, which corresponded to our previous findings in spring wheat [21], the remaining loci were identified in winter wheat.

The MTAs for gluten content identified in a collection of spring wheat varieties and introgression lines with substitutions and translocations from species of tribe *Triticeae* (*T. durum*, *T. dicoccum*, *T. dicoccoides*, *T. timopheevii*) also overlapped with loci previously reported in both spring and winter wheat. For example, the SNP on chromosome 1D (wsnp_Ex_c35886_43950102), the three-SNP locus on the short arm of chromosome 2A (wsnp_BE498730A_Ta_2_2, Tdurum_contig45580_1717, Excalibur_c21269_176), the SNPs on chromosome 2B (RAC875_c14105_66 and RFL_Contig3016_1091), and the SNP on chromosome 5A (wsnp_Ex_c4921_8764106) were located within or close to loci reported in winter wheat [42,45]. The SNP RAC875_rep_c112818_307 on chromosome 5A was part of three previously described loci, one identified in winter wheat and two in spring wheat [38,45]. In addition, the locus on the short arm of chromosome 7B was located near a locus reported in spring wheat [40].

A comparative analysis of loci identified in winter and spring wheat revealed largely overlapping genomic regions, suggesting that the genetic control of grain protein content is broadly shared between varieties of this two growth habits. However, quantitative differences in allelic effects or gene expression were not statistically evaluated in this study due to the limited availability of comparable published data. Most GWAS and QTL studies do not specify genes within associated loci or provide expression data, and comprehensive transcriptomic datasets across developmental stages and tissues are still scarce. Therefore, direct comparisons of allelic effects or gene expression patterns between winter and spring genotypes are currently not feasible.

### 3.2. Candidate Genes for Stable Loci

To identify candidate genes potentially associated with grain protein content, we selected three loci with high confidence that, in addition to our work, were reported in at least two other studies. These loci were located on the long arms of chromosomes 3D and 5A and on the short arm of chromosome 6A. Since genes influencing grain protein content are predominantly expressed in the upper and flag leaves after flowering or directly in the developing grain [17,59], candidate genes were chosen based on preferential expression in these tissues. In total, five genes from chromosome 3D, one gene from chromosome 5A, and nine genes from chromosome 6A were identified (Table 2). All of these genes are poorly characterized, and only preliminary bioinformatic annotations are currently available, making it difficult at this stage to predict the mechanisms through which they may contribute to the regulation of grain protein content in common wheat.

Since no direct effect of the identified candidate genes on grain protein content was established, we further explored their potential functional associations using the KnetMiner platform. Through this analysis, we identified the following relationships: *TraesCS6A02G103300* was associated with *Vp1-3A* (*TraesCS3A02G417300*), *TraesCS6A02G130300* with *Vp1-3D* (*TraesCS3D02G412800*), and *TraesCS6A02G132700* with *Vp1-3B* (*TraesCS3B02G452200*).

The *Vp1* genes in wheat are well known for their association with pre-harvest sprouting resistance [60]. In addition, they have been linked to seed maturation processes, which may involve the accumulation of various storage compounds in the grain [61]. In maize, D. McCarty et al. (1991) demonstrated that *Vp1* plays a central role in coordinating multiple developmental pathways during seed formation [62]. Moreover, *Vp1* was shown to be essential for scutellum development and for the transfer of proteins from the endosperm to the embryo [63]. In *vp1* mutant embryos, scutellum cells are misshapen and contain significantly less cellular material, disrupting normal protein accumulation. Evidence also indicates that *Vp1* transactivates genes involved in sulfur assimilation and nutrient metabolism, thereby directly influencing grain protein content [63].

Another interesting relationship revealed by KnetMiner involves the gene *TraesCS3D02G538000*, which appears to be indirectly associated with starch content in the endosperm through *NAC019-3A*. The *NAC019-3A* gene regulates starch synthesis in the wheat endosperm [64]. As previously reported, starch content in wheat grains inversely affects protein concentration due to a dilution effect, whereby increased starch accumulation leads to a proportional decrease in protein percentage [65]. Hao et al. (2022) empirically confirmed this relationship, showing that an increase in starch content was accompanied by a simultaneous decrease in grain protein content [66]. Similarly, Zib et al. (2025) further substantiated this pattern, noting that grain protein and starch concentrations exhibit opposite trends during grain development [67]. Overall, the evidence supports a robust and consistent dilution effect, suggesting that *TraesCS3D02G538000* may influence grain protein content indirectly—by modulating its ratio to starch accumulation rather than through direct regulation of protein synthesis.

## 4. Materials and Methods

### 4.1. Plant Material

The plant material used in this study was a collection of winter common wheat (*Triticum aestivum* L.) varieties provided by the N.I. Vavilov All-Russian Institute of Plant Genetic Resources. This collection consisted of 170 wheat varieties from various regions of the world, including 96 varieties developed through Russian breeding programs. A detailed description of the collection has been published previously [68]. To assess protein and gluten content, the collection samples were grown in a randomized block design with two replicates on 1 m^2^ plots in 2021 and 2022 in the Oryol Region (52°51′ N, 36°26′ E). The data of temperature and precipitation for the two environments are presented in Supplementary Table S7. The soil type is dark gray forest, heavy loam. The humus content is 4.71%, which corresponds to a medium level. The soil is moderately acidic (pHHCl–4.9). The contents of available phosphorus and potassium are 225.8 mg/kg (high) and 112.2 mg/kg (medium), respectively.

### 4.2. Evaluation of Protein and Gluten Content

Protein and gluten content in the grain was measured using an OmegAnaLyzer G (Bruins Instruments, Munich, Germany) infrared grain analyzer (730–1100 nm). To determine the percentage of protein and gluten, whole grain samples were placed in a sliding cuvette with a volume of 10 g; the measurement time was 30 s. Each variety was measured in triplicate, with grain replaced between replicates. Moisture content was also measured using the OmegAnaLyzer G (Bruins Instruments, Munich, Germany), which allowed for subsequent recalculation of the values on a dry weight basis (Table S1).

### 4.3. DNA Extraction

Genomic DNA was extracted using a modified sodium bisulfite protocol as described by [69]. DNA samples intended for SNP genotyping were purified with the Bio-Silica DNA Purification Kit (Bio-Silica, Novosibirsk, Russia) according to the manufacturer’s instructions. The purified DNA was quantified using the Qubit™ dsDNA BR Assay Kit (Thermo Fisher Scientific, Waltham, MA, USA) on a Qubit™ 4 Fluorometer (Thermo Fisher Scientific).

### 4.4. Analysis of NAM-1 Genes Alleles

To determine the alleles of the *NAM-B1* gene in the panel of winter wheat varieties, primers described by Yang et al., 2018 [70] were used: NAMB1MYF (5′-CCCCGGGCTAGGTACAAAGGT-3′), NAMB1MYR (5′-AATTTGCGGCGCTTGATAAAG-3′), and NAMB1R2 (5′-GGGACTACCACAAACTGCAACAG-3′). To assess the allelic variation in the *NAM-A1* gene, published KASP markers were used [34]. All PCR primers were synthesized at LGC Genomics (Hoddesdon, UK). Each reaction mixture consisted of a final volume of 10 µL containing 5 µL template DNA (100–150 ng), 5 µL V4.0 2 × Mastermix (LGC, BiosearchTechnologies, Hoddesdon, UK), 0.14 µL primermix (12 µM each allele-specific primer and 30 µM common primer). The PCR reaction was performed on a QuantStudio™ 5 real-time PCR system (Thermo Fisher Scientific, Foster City, CA, USA) under the following cycling conditions: denaturation at 94 °C for 15 min; 10 cycles of 94 °C for 20 s and touchdown starting at 62 °C for 1 min (decreased by 0.6 °C per cycle); and 26 cycles of amplification (94 °C for 20 s; 57 °C for 1 min). Data analysis was performed automatically by QuantStudio™ Real-Time PCR Software v1.3 (Applied Biosystems, Foster City, CA, USA).

### 4.5. Statistical Analysis

Analysis of variance (ANOVA) was performed using the R function aov. A two-way ANOVA was conducted to evaluate the significance of differences among genotypes and environments, with genotypes treated as a fixed effect and environments and replications as random effects. Spearman’s correlation coefficients (r^2^) were calculated with the R function cor. The Shapiro–Wilk test was used to assess the normality of trait distributions. To estimate trait heritability, a linear model with all effects considered as random was fitted using the R package lme4 (version 1.1-37) [71]. Broad-sense heritability (H^2^) was then calculated using the standard formula for heritability estimation (“standard” broad-sense heritability method) as described by Holland, Nyquist & Cervantes-Martínez [72]. The broad-sense heritability (H^2^) across all environments was estimated using the following formula:

$$H^{2}=\;\frac{\sigma_{G}^{2}}{\sigma_{G}^{2}\;+\;\left(\frac{\sigma_{G*E}^{2}}{n_{E}}\right)\;+\left(\frac{\sigma_{e}^{2}}{n_{E\;}+\;n_{R}}\right)}$$

where $\sigma_{G}^{2}$, $\sigma_{G*E}^{2}$, $\sigma_{e}^{2}$ represent the genotypic, genotype-by-environment interaction, and residual (error) variances, respectively. $n_{E}$ and $n_{R}$ denote the number of environments and the number of replications within each environment, respectively.

### 4.6. Genome-Wide Association Study (GWAS)

High-throughput SNP genotyping was performed using the Illumina Infinium platform (Illumina Wheat 25K SNP chip, which contains 24145 SNP markers in total) at TraitGenetics GmbH (Gatersleben, Germany). A total of 20,008 polymorphic SNP markers were selected for further analysis after filtering based on minor allele frequency (MAF > 0.1) and a minimum call rate of 90% across the population.

Genome-wide association analysis was conducted using the GAPIT3 R package (version 3.5) [73]. For each trait, several statistical models were tested: the Mixed Linear Model (MLM) and the Compressed Mixed Linear Model (CMLM), both of which accounted for population structure (PCA) and kinship matrix (calculated using the “VanRaden” method), as well as the BLINK and FarmCPU models. The best-fitting model was selected based on quantile–quantile (QQ) plots. Manhattan and QQ plots were generated using the R package CMplot (version 4.5.1) [74].

The chromosomal positions of SNPs and genes within the associated loci were identified using the RefSeq v1.1 wheat genome assembly available through the GrainGenes genome browser [75].

### 4.7. Candidate Genes

Gene expression patterns for candidate loci were evaluated using the expVIP database [76] to select those predominantly expressed in grain as well as in flag and fifth leaves after anthesis. Functional annotation of genes was performed using the Persephone genome browser [77]. Potential links to phenotypes were determined using KnetMiner [78].

### 4.8. Comparison with Previously Published Data

Data were compiled from 17 publications (Table S4) in which markers were either reported with their physical positions on the RefSeq map or could be aligned to the reference genome through available sequences or positional information in databases or other studies.

## 5. Conclusions

Thus, a comparative analysis of the data we obtained in this and a previous study and presented in the literature on the genetic control of protein and gluten content in wheat grains indicated that genetic loci and associated molecular markers did not significantly differ between spring and winter wheat. The identification of specific markers associated with protein content largely depended on the particular sample set. Despite differences in growing conditions and the mechanisms of protein accumulation during development and maturation, we proposed that the genetic mechanisms determining grain protein content in common wheat do not depend on wheat growth habit. Based on these findings, we propose that breeding programs may benefit from transferring high-GPC alleles and loci identified in spring wheat into winter backgrounds. This approach would combine the favorable protein alleles of spring types with the adaptive and yield-related traits of winter types. Our future research will focus on integrating yield-related traits with grain protein and gluten content to identify genotypes combining high yield potential with superior grain quality.

## Figures and Tables

**Table 1:** SNP markers associated with grain protein and gluten content in winter wheat varieties, identified using a linear mixed model accounting for population structure and kinship matrix. The most significant markers are shown in bold. * Effector alleles are shown in bold.

SNP	Chr	Position, RefSeq v1.1	Position, RefSeq v2.1	Alleles *	Protein *p*-Value	Protein Effect	Gluten *p*-Value	Gluten Effect
Kukri_c31121_1460	2A	738 990 026	610 373 521	**C**/T	0.0009	0.84		
AX-158540499	2A	746 754 847	750 756 090	**G**/A	0.0007	0.84		
**Excalibur_c44325_339**	2D	608 198 980	611 140 887	**C**/A	0.00003	1.13	0.0002	2.79
AX-158523608	3A	711 297 651	711 880 200	**G**/A	0.0006	−0.84	0.0005	−2.31
AX-110989461	3A	711 693 497	712 274 084	**T**/C	0.0006	−0.83	0.0005	−2.28
wsnp_Ex_c361_707953	3A	732 471 093	733 780 140	**A**/G	0.001	0.80	0.0006	2.24
wsnp_Ex_c1097_2105209	3B	82 447 575	94 202 166	**G**/A			0.0005	−1.89
AX-110941989	3B	96 834 446	108 604 382	**T**/C			0.0008	−1.80
AX-95109644	3B	116 115 703	127 844 424	**T**/C			0.0005	−1.88
Excalibur_c17654_1090	3D	611 298 383	615 380 128	**T**/C	0.0008	0.70		
**wsnp_Ex_c21197_30325539**	4A	544 177 849	543 159 477	**C**/T	0.00004	1.11	0.00002	3.15
**AX-158533442**	4A	544 178 037	543 159 713	**C**/T	0.0002	1.04	0.00005	3.08
**AX-158524430**	4A	544 389 263	543 368 444	**G**/C	0.0003	1.07	0.0001	3.14
**BS00109736_51**	4A	544 616 571	543 598 448	**A**/G	0.0001	1.14	0.00004	3.29
**IAAV6944**	4A	544 616 659	543 598 559	**G**/A	0.0002	1.06	0.0001	3.11
**wsnp_Ex_c24443_33687802**	4A	544 891 219	543 871 484	**C**/T	0.0005	1.03	0.0002	3.02
**wsnp_Ex_c24443_33688235**	4A	544 891 555	543 871 917	**C**/T	0.0005	1.03	0.0002	3.02
**AX-158598641**	4A	545 113 750	544 093 204	**C**/T	0.0003	1.06	0.0001	3.23
**TA001512-0387**	4A	545 601 781	544 578 398	**A**/G	0.0002	1.09	0.0001	3.16
**Ra_c37920_342**	4A	545602051	544 578 621	**T**/C			0.0002	2.99
**BobWhite_rep_c65013_174**	4A	545 603 625	544 580 195	**C**/T	0.0008	0.99	0.0001	3.08
**AX-158581338**	4A	545 618 766	544 595 359	**G**/A	0.0006	1.02	0.0001	3.08
Kukri_c48199_102	4B	78021175	81058096	**G**/A			0.0005	2.49
Excalibur_c874_1479	5A	580 946 185	569 913 748	**G**/A	0.001	0.76		
AX-110033497	5B	31017228	31392648	**C**/T	0.0006	−0.78		
wsnp_Ex_c8962_14947544	5B	31114116	31489586	**G**/A	0.0006	−0.78		
RAC875_rep_c102342_470	5B	573495707	576702400	**A**/G	0.0004	−0.96	0.0005	−2.54
TA001138-0446	5B	585754057	589173932	**A**/G	0.0005	0.71	0.0005	1.92
AX-94647124	6D	9 358 293	10 415 129	**A**/G	0.0003	1.21	0.0004	3.20
AX-158567766	7B	625885621	631366598	**T**/C	0.0006	−0.64		
BS00089942_51	7B	626239215	631718537	**A**/G	0.0008	−0.65		

**Table 2:** List of candidate genes for grain protein content and their functional description, extracted from the most stable genomic loci detected in three or more independent studies.

Name	Function Description	InterPro ID	PFAM ID
*TraesCS3D02G534600*	Ankyrin repeat-containing protein	IPR002110; IPR020683; IPR026961	
*TraesCS3D02G538000*	Protein CHUP1, chloroplastic		
*TraesCS3D02G538600*	Pectin acetylesterase		PF03283
*TraesCS3D02G541400*	Regulator of chromosome condensation (RCC1) family with FYVE zinc finger domain-containing protein		
*TraesCS3D02G542900*	Dirigent protein	IPR004265	PF03018
*TraesCS5A02G430400*	ATPase subunit 8	IPR003319; IPR009455	PF02326; PF06449
*TraesCS6A02G093200*	Endoglucanase	IPR001701; IPR008928; IPR018221	PF00759
*TraesCS6A02G099600*	Mitogen-activated protein kinase	IPR000719; IPR003527; IPR008271; IPR011009; IPR017441	PF00069
*TraesCS6A02G102800*	F-box family protein	IPR001810	
*TraesCS6A02G103300*	Glycine rich protein		
*TraesCS6A02G130300*	Anthocyanin 5-aromatic acyltransferase	IPR003480	PF02458
*TraesCS6A02G132700*	SNF1-related protein kinase regulatory subunit gamma-like PV42a	IPR000644	PF00571
*TraesCS6A02G135600*	Leucine-rich repeat receptor-like protein kinase family protein	IPR001611; IPR003591; IPR013210; IPR032675	PF08263; PF13855; PF00560
*TraesCS6A02G135800*	Leucine-rich repeat receptor-like protein kinase family protein, putative	IPR001611; IPR003591; IPR013210; IPR032675	PF08263; PF13516; PF13855
*TraesCS6A02G136100*	Leucine-rich repeat receptor-like protein kinase family protein, putative	IPR001611; IPR003591; IPR013210; IPR025875; IPR032675	PF08263; PF13855; PF12799

## Data Availability

All the data presented in this study are included in the manuscript and Supplementary Materials.

## Supplementary Materials

The following supporting information can be downloaded at: https://www.mdpi.com/article/10.3390/ijms262211159/s1.

## Abbreviations

The following abbreviations are used in this manuscript:

GPC	Grain protein content
GC	Gluten content
GWAS	Genome-wide association studies
QTLs	Quantitative Trait Loci
MTAs	Marker–trait associations
SNP	Single-nucleotide polymorphism
MLM	Mixed Linear Model
PCA	Principal component analysis
