# Supplementary material for: Genetic Control of Grain Protein and Gluten Content: Winter vs. Spring Wheat
Source: Int J Mol Sci. 2025 Nov 18;26(22):11159. doi: 10.3390/ijms262211159 (PMC12653508; doi:10.3390/ijms262211159)

Figure S1. Scatter plot showing the correlation between protein and gluten content based on mean values. The Spearman correlation coefficient and significance level are shown in the top left corner. The significance level indicated by *** corresponds to p-values < 0.0001.

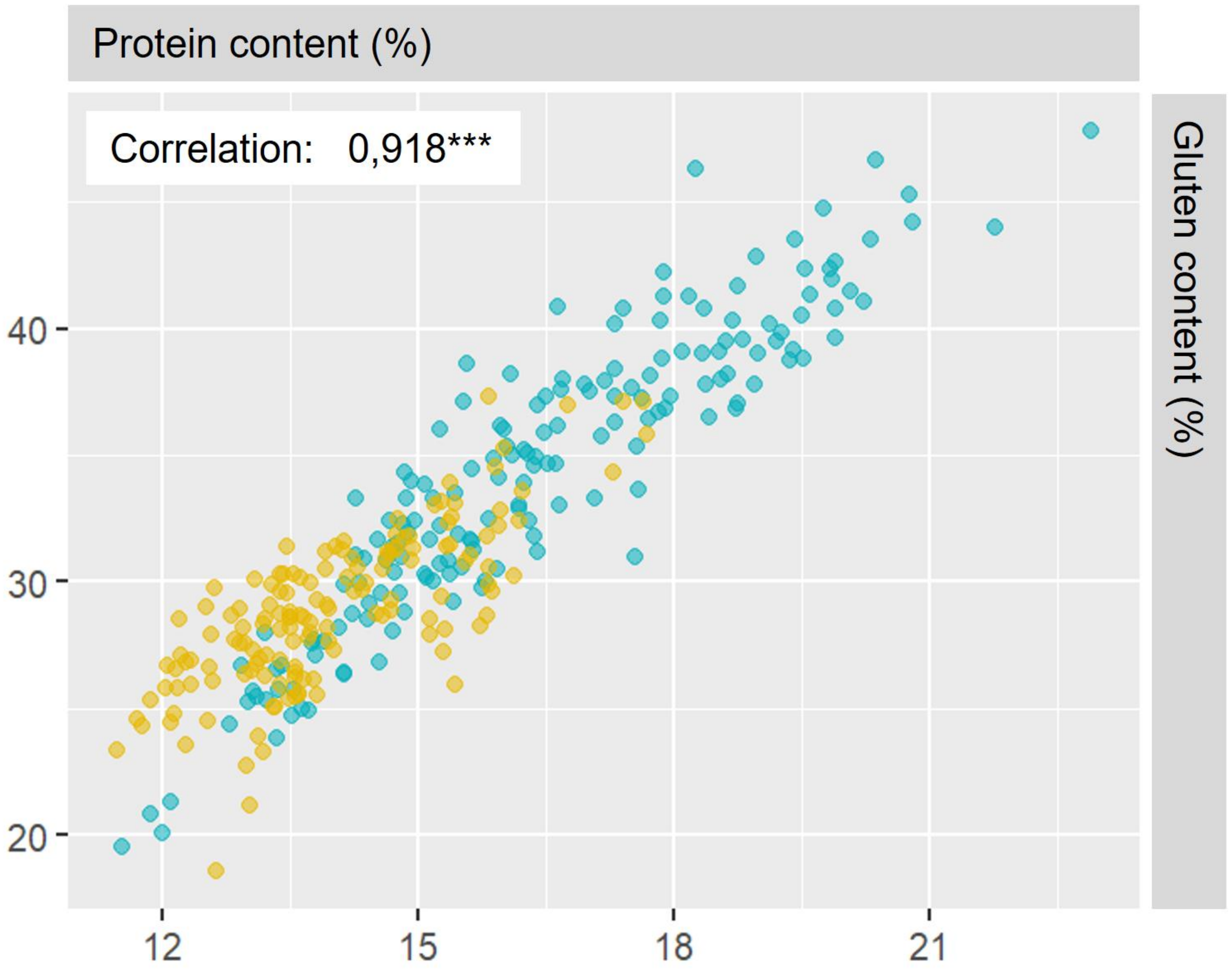

Figure S2. Allelic distribution of the *NAM-A1* gene identified using KASP markers in a population of winter common wheat cultivars.

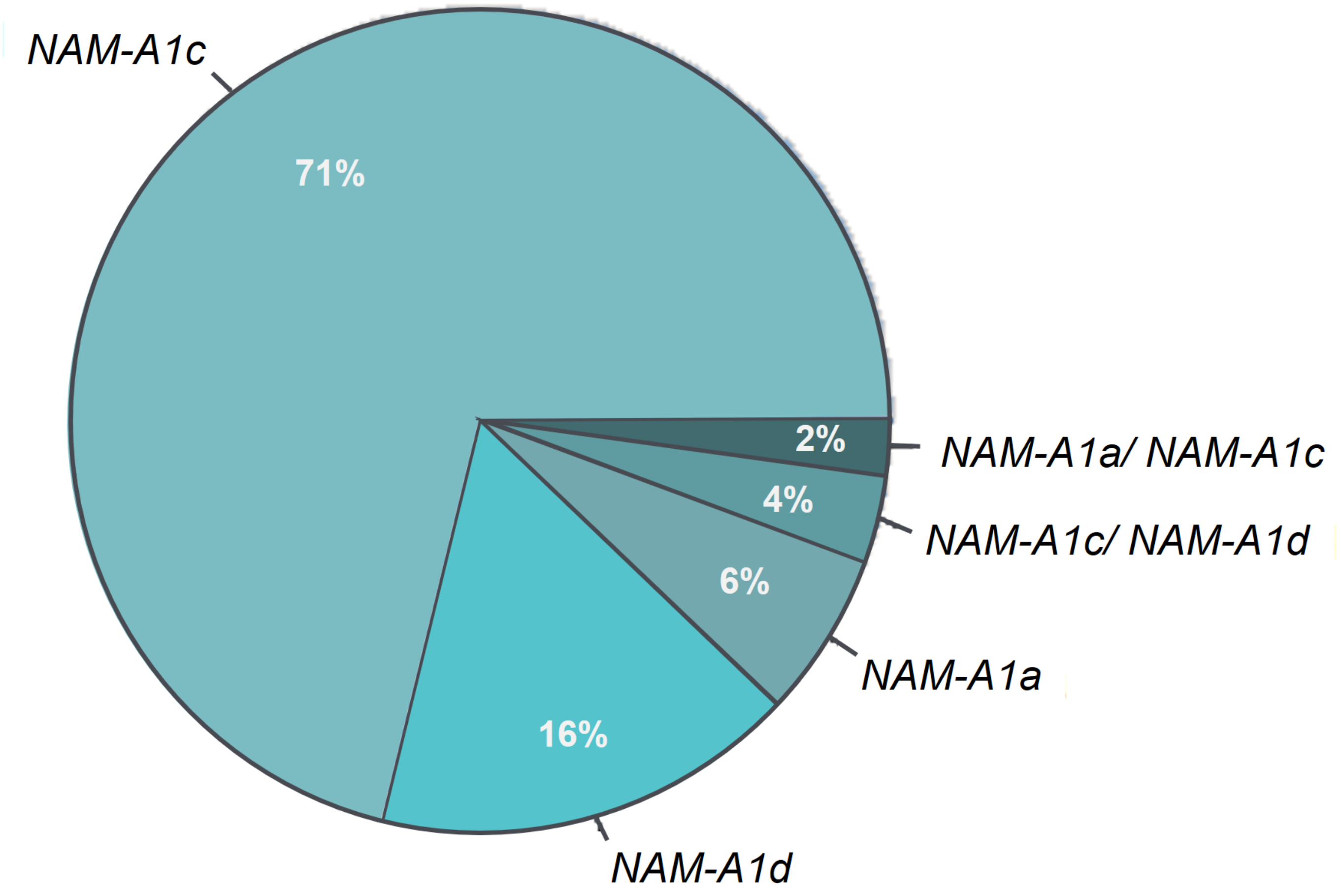

Figure S3. Localization of GPC-associated QTLs. Visualization is performed using Persephone resource (https://web.persephonesoft.com).

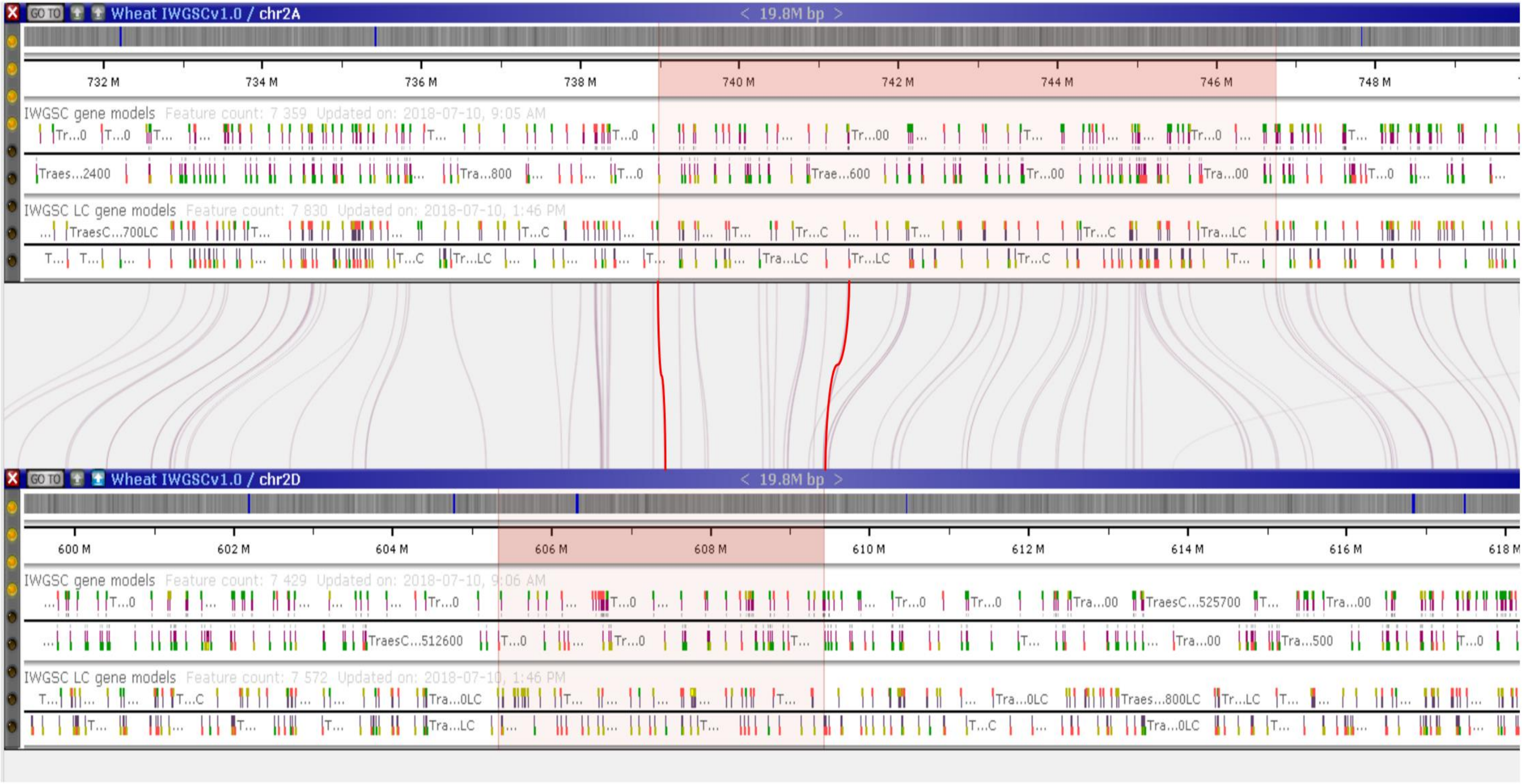

Supplement: Supplementary file 1 [file ijms-26-11159-s001.zip › Supplementary_Figures S1-S3.pdf]
